# Comparison of the Complications between Left Side and Right Side Subclavian Vein Catheter Placement in Patients Undergoing Coronary Artery Bypass Graft Surgery

**DOI:** 10.15171/jcvtr.2014.003

**Published:** 2014-09-30

**Authors:** Masoud Tarbiat, Babak Manafi, Maryam Davoudi, Ziae Totonchi

**Affiliations:** ^1^Ekbatan Cardiovascular Surgery Center, Department of Anesthesiology, Hamedan University of Medical Sciences, Hamedan, Iran; ^2^Department of Anesthesiology, Rajaei Heart Hospital, Iran University of Medical Sciences, Tehran, Iran

**Keywords:** CABG, Subclavian Vein Catheter, Infraclavicular Approach

## Abstract

*Introduction:* Percutaneous subclavian vein catheterization is one of the most common invasive procedures performed in cardiac surgery. The aim of this study was to compare left and right subclavian vein catheter placement via the infraclavicular approach in patients who undergo coronary artery bypass graft (CABG) surgery.

*Methods:* This prospective, randomized clinical trial was performed in193 patients. The technique applied for cannulation was infraclavicular approach for both the right and the left sides. Subclavian vein of other side was attempted only when catheterization at initial side was unsuccessful at two attempts. The success and complication rates were compared for the two sides.

*Results:* On193 patients, catheterization attempts were performed. Overall 177 catheterizations (91.7%) were successful during the first attempt, 105 (92.1%) on the right side and 72 (91.1%) on the left side. There was no significant difference between success rate and side of catheterization. Malposition of the catheter tip on the right side (9.6%) was significantly more than the left side (0%) (P= 0.003). The differences in other complications on two sides were statistically insignificant.

*Conclusion:* Compared with the right side, insertion of the cannula on the left side resulted in fewer catheter tip misplacements. Incidence of cannulation failure and other complications were similar on both sides.

## Introduction

Percutaneous subclavian vein catheterization is one of the most common invasive procedures performed by anesthesiologist in cardiac surgery.

Advantages of subclavian venous cannulation in comparison with internal jugular and femoral approaches include a lower risk of infection and increased patient comfort especially for long-term intravenous therapy.^1,2^ This catheter is commonly inserted for central venous pressure monitoring, administration of medications, rapid infusion of fluids (via large cannulas), cardiopulmonary resuscitation, insertion of a transvenous pacemakerand difficult peripheral catheterization.^3,4^ Infraclavicular approach to the subclavian vein was first described by Aubaniac in 1952 and has been well reviewed by Defalque in1972.^3^

This is a blind procedure and requires localization of a deep vein using only superficial anatomical landmarks.^5^ Because of the blind nature of the procedure, it can be extremely difficult in some patients to localize the vein. Thus, the procedure frequently requires multiple needle attempts. In reported series, the complication rates range from 3% to12%, depending on the experience of the persons performing the procedure and definition of a complication.^6-9^

These studies also showed that history of a prior surgery or radiotherapy in the region of the subclavian vein may affect the rate of complications.^6,9^

In more practiced hands, the incidence of complications should be low, pneumothorax developing in less than 2% and arterial puncture in less than 5% of cases.^6^ The potential problems and complications associated with attempted subclavian vein catheterization include failure to locate and exactly cannulate the vein, catheter malposition, pneumothorax, subclavian artery puncture, hemothorax and brachial plexus or other nerve injury.^6^ These complications increase patient’s morbidity, prolong hospitalization or delay therapy. The most common complication is failure to cannulate the subclavian vein.^9^ Malatinsky and colleagues noticed a 5.5% incidence of misplaced catheters with the infraclavicular approach, all of which occurred from right-sided approach.^10^

The high incidence of misplaced catheters from the right side was confirmed by Padberg et al.^11^ Although the relationship between the side and incidence of other technical complications was not mentioned in either study.

There are numerous anatomical differences between left and right sides of subclavian vessels. On the right, the subclavian-jugular venous junction overlies the subclavian artery, making this vessel more prone to injury than it is on the left.^12^

Perhaps to an even greater degree than with internal jugular vein cannulation, the safety of subclavian venous line placement rests in the experience of the operator.^13^

Malposition of central venous catheter is a known complication, when inserted into subclavian vein. A malpositioned catheter tip gives inaccurate central venous pressure readings. Thrombosis or thrombophlebitis of the internal jugular vein, retrograde perfusion of the intracranial veins are other complications which may occur. Fear of infection remains if repositioning of the catheter is attempted.^14^ We carried out this prospective study on left and right side infraclavicular subclavian catheterizations to compare the incidence of failure, and the incidence of technical complications on the both sides.

## Material and methods

Between October 2012 and July 2013, this prospective, randomized clinical trial was performed at Hamedan University of Medical Sciences, to compare left and right subclavian vein catheters displacement in patients undergoing coronary artery bypass graft (CABG ) surgery with harvesting the left internal mammary artery (LIMA).

Over a nine-month period, 193 patients under CABG surgery with harvesting LIMA were enrolled in this study. All patients were randomized selected as L (left) or R (right) side for catheterization.

Our exclusion criteria were emergency surgery, prior radiotherapy at the attempted catheterization site, abnormalities in the platelet count or coagulation times, prior surgery within the region of subclavian vein catheterization (e.g. mastectomy, axillary node dissection, or thoracotomy).

The procedure was done after induction of anesthesia and tracheal intubation. Before the procedure, the patient was placed in the Trendelenburg position (15 to 30 degrees head down) to avoid an air embolus and to distend the subclavian vein. The patient’s head was turned slightly to the contralateral side, and the arm was kept in the side.

Before putting on surgical hand gloves, a spot (1 cm) was marked caudal to the clavicle at the junction of the middle and medial thirds of the clavicle, then the subclavian area was prepared and disinfected.

An 18 gauge needle was used on a 5 ml syringe, aspirating as the needle was advanced. As subclavian vein lies deep to the clavi-pectoral fascia and the resistance of the fascia was easily appreciated when it was pierced. Plunging into the vein was marked by a flush of blood.

As needle was stabilized with the thumb and forefinger, the syringe was removed and the hub of the needle was immediately occluded (maintaining a “closed system”) and threaded the J-wire into the 18 gauge needle leaving about half of the wire extruding from the needle.

Then the J-wire was secured with a fingertip and removed the 18 gauge needle over the exposed, remaining portion of the J-wire, and made a small cut in the skin adjacent to the entry site of the J-wire using a scalpel.

The silastic dilator was threaded over the J-wire and advanced the dilator fully into the chest, and removed the dilator while still leaving the J-wire in place.

The hub was removed from the long central catheter (Tri-lumen ARROW central catheter; 20 cm), and threaded the long central catheter over the J-wire into the vein. All central catheters threaded less than 15 cm length into the vein. At the end, 5 to 10 cm of the catheter was left outside the skin, the J-wire was carefully removed, and attached intravenous tubing to the catheter, and finally catheter was sutured to the skin.

After the catheter was placed, a chest x-ray was taken to evaluate for complications in the ICU (Pneumothorax, hemothorax, and misplacement of the catheter). Procedures were monitored for arterial puncture, evidence of LIMA injury, and accurate recording of the number of passes. Because of harvesting the LIMA, all patients had chest tubes in the left hemithorax. The choice of which side to cannulate was the patients’ choice by choosing a L or R sheet randomizely. The 114 right-sided insertions were compared with the 79 left-sided.

The cannulation was undertaken by clinicians experienced in its use. (i.e. an experience of greater than 25 insertions on a month at least for 5 years). All clinicians were right-handed. Pneumothorax was treated with anterior intercostal underwater seal drains when necessary, and subclavian arterial punctures were treated with five minutes of digital pressure over the insertion site after removal of the needle.

Variables study: age, sex, height, weight, body mass index (BMI), the number of needle passes (defined as separate skin punctures) attempted, the side on which the catheter insertion was attempted, and probable complications such as malposition, pneumothorax, hemothorax, subclavian arterial puncture, injury of LIMA, and thoracic duct damage. Routine evaluation prior to subclavian vein catheterization included a chest X-ray, platelet count, prothrombin time (PT), and partial thromboplastin time (PTT). Every patient had LIMA graft on LAD (left anterior descending) artery, and chest tube in the left hemithorax.

The Chi-Square test was used to assess the relationship between catheterization procedure success rate and the cannulation side. It was also used to determine the relationship between the cannulation side and the complications. The P value less than 0.05 indicated statistical significance. All statistical calculations were performed using SPSS version 19 software.

## Results

There were 193 patients of mean age 58.2 years (range 33-82 years). The lowest BMI was 16.7 and the highest BMI was 40(Table 1). Infraclavicular approach catheterization was attempted on the right side subclavian vein in 114 patients and on the left side in 79 patients. In 177 (91.7%) patients the first attempt at subclavian catheterization was successful, 105 (92.1%) on the right side and 72 (91.1%) on the left side. In 9 patients, the second attempt on subclavian catheterization was successful, 3 (2.6%) on the right side and 6 (7.6%) on the left. The overall success rate in two attempts were 108 (94.7%) on the right side and 78 (98.7%) on the left side. After second attempt, 6 right-sided attempts and 1 left-sided attempt failed and were subsequently successful on the other sides. On the opposite side, three (2.6%) attempts was successful on the initial right side unsuccessful attempts and 1(1.3%) on the initial left side unsuccessful attempt. Three (1.6%) attempts at infraclavicular subclavian catheterisation failed on the both sides, all of these were initially unsuccessful on the right side(Table 2).

**Table 1:** Patient Characteristics

**Characteristic**	**Right**	**Left**	**P-value**
Sex ratio, Male/Female	75/39	45/34	0.23
Mean age, years ( Mean ± SD )	58.14±10.8	58.3±9.82	0.91
Weight, Kg ( Mean ± SD )	68.14±12.42	67.72±12.37	0.81
Height, Cm ( Mean ± SD )	159.2 ± 12.05	159.67 ± 9.1	0.74
BMI, Kg/m^2^ ( Mean ± SD )	28.66 ±4.94	26.64± 4.87	0.97
BMI < 20 Kg/m^2^	6 (50%)	6 (50%)	0.89
BMI 20-25 Kg/m^2^	46(61.4%)	32(38.6%)	
BMI 25-30 Kg/m^2^	39(59.1%)	27(40.9%)	
BMI > 30 Kg/m^2^	26(57.8%)	19(42.2%)	

**Table 2:** Relationship between side of catheterization and success rate

Side Success rate	**Right side (%)**	**Left side (%)**	**Total (%)**
Success	111 ( 97.4%)	79(100%)	190 (98.4%)
Failure	3(100%)	0 (0%)	3(1.6%)
Total	114(100%)	79(100%)	193(100%)

(P= 0.271)

Finally a central venous line was subsequently inserted in right internal jugular vein. There were no significant differences between success rate and side of catheterization.

Pneumothorax, subclavian arterial puncture, and malposition of the catheter tip were major complications observed (Table 3).

**Table 3:** Complications between right and left subclavian catheterizations

	**Right**	**Left**	**P-value**
Malposition			
Ipsilateral Internal Jugular Vein	10	0	0.003
Contralateral Subclavian Vein	1	0	
Pneumothorax	5	0	NS
Subclavian artery puncture	2	1	NS

NS: No Significant; P> 0.05

**Figure 1:**
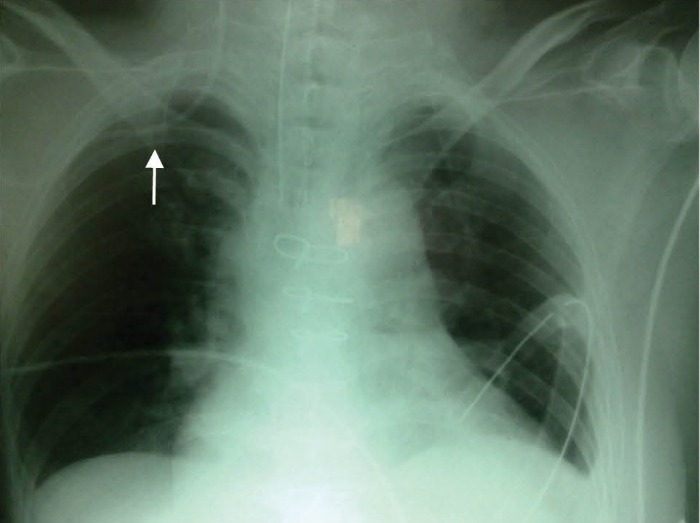
Post-operative chest x-ray depicting right subclavian vein catheter malpositioned into ipsilateral internal jugular vein (arrow)

**Figure 2:**
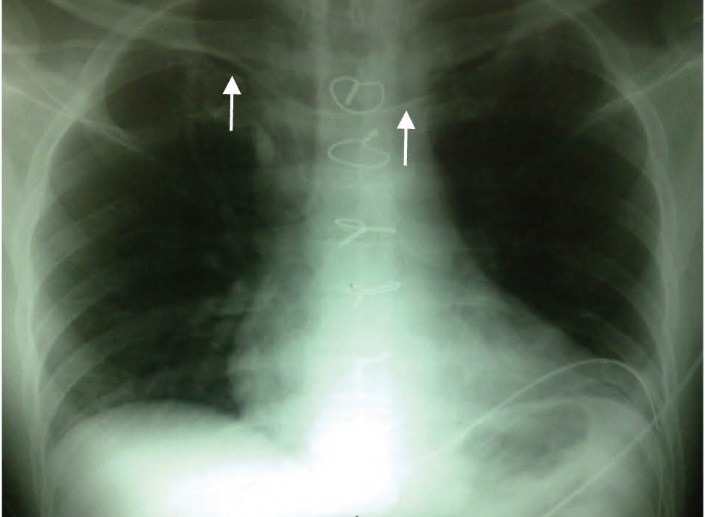
Post-operative chest x-ray depicting right subclavian vein catheter malpositioned into contralateral subclavian vein (arrows)

Malposition of the catheter tip when inserted on the right side (9.6%) was significantly more common than on the left side (0%) (*P*= 0.003). In ten patients, the catheter tip was located in the ipsilateral internal jugular vein, (Figure 1) and in one, the catheter tip was located in the contralateral subclavian vein (Figure 2).

The overall malposition of the catheter tip on both sides was 5.7%.‏ In 5 patient, Pneumothorax was confirmed by chest x-ray. Pneumothorax occurred only in patients with right side catherization (4.3%). Subclavian arterial puncture occurred in 3 patients, 2 in right-sided attempts and 1 in left-sided attempt. The incidence rate was 1.6%.

There was no significant difference between success rate and side of catheterization** (**Table 2**).** There was no injury of thoracic duct or hemothorax on both sides of catheterization.

## Discussion

The subclavian vein is an extension of the axillary vein at the outer border of the first rib. It runs under the clavicle, where they connect to the internal jugular veins to form the innominate, or brachiocephalic veins.

Central venous cannulation using the infraclavicular approach to the subclavian vein has a greater incidence of technical complications when compared with internal jugular vein cannulation or supraclavicular subclavian cannulation.^14^

Mc Goon and colleagues listed 23 reported complications with subclavian cannulation whereas they found only 16 reported complications with internal jugular vein cannulations.^15^ The incidence of complications however was not related to the side of cannulation.

Fischer et al. suggested that right-sided subclavian cannulation may reduce the complications associated with this procedure, reasoning that the cupola of the lung is lower and the thoracic duct is on the opposite side.^16^

In our study, there was no significant difference between pneumothorax and the side of catheterization, although it only occurred with the right side catheterization because all patients had chest tube in the left hemithorax.

Arterial puncture and perforation during subclavian cannulation appears to be mostly a right sided phenomenon, which coincides with the anatomic differences of the vascular system at either side of the midline. On the right, the subclavian-jugular venous junction overlies the subclavian artery, making this vessel more prone to injury than it is on the left.^12^ We found no significant difference between subclavian arterial puncture and side of catheterization.

Bold et al. reported a 69.7% initial success rate (1 or 2 attempts) for subclavian vein catheter insertion.^9^

In Kilbourne at al. study, Surgical and emergency medicine residents had a 89.5% success rate for subclavian vein catheter insertion.^17^ In our study, initial success rate (1 or 2 attempts) was 96.4%. This difference may be due to clinicians’ experiences (i.e. an experience of greater than 25 insertions on a month at least for 5 years). There was no significant difference between success rate and side of catheterization. It should be noticed that the tip of catheter is inserted to the just above the junction of the superior vena cava and the right atrium.

Conces and colleagues have reported proper position in 68% of successful catheter placements.^18^

Matthews et al. reported 15% malposition of the catheter tip when inserted on the right side, significantly more common than malposition of the catheter when inserted on the left side (2%).^14^ In present study too, malposition of the catheter tip on the right side (9.6%) was significantly more common than the left side (0%). The overall malposition of the catheter tip on both sides was 5.7%.We found similar incidence of failure of catheter insertion and complications. However, we observed no catheter tip malposition on the left side when compared with the right side which may be due to anatomic differences of the vascular system at either side of the midline. The right subclavian vein enters the innominate at a sharper angle than its counterpart on the left.^12^

## Conclusion

Result of this study suggests that in case of infraclavicular approach to subclavian cannulation during CABG surgery with harvesting the LIMA, left sided cannulation of the subclavian vein is a better option.

## Acknowledgements

The authors would like to thank Dr. Manzar Hussain Akbar for review and editing this manuscript. This study was approved and supported by Hamedan University of Medical Sciences.

## Ethical Issues

This study was reviewed and approved by the research center of Hamedan University of Medical Sciences and registered in IRCT (Iranian Registry of Clinical Trials). All patients participating in this study provided written informed consent and all data were collected by a single nurse (Trial registration: http://www.irct.ir; identifier: IRCT2012072110348N1).

## Competing interests

Authors declare no conflict of interest in this study.
